# Being a Radiation Oncologist in Algeria

**DOI:** 10.7759/cureus.96398

**Published:** 2025-11-08

**Authors:** Asma Mous, Salim Chaib Rassou, Aicha D Benddjazia, Layth Mula-Hussain

**Affiliations:** 1 Department of Radiotherapy, Faculty of Medicine, Dr. Benaouda Benzerdjeb, Abou Bekr Belkaid University of Tlemcen, Tlemcen, DZA; 2 Department of Radiation Oncology, Sultan Qaboos Comprehensive Cancer Care and Research Centre (SQCCCRC), Seeb, OMN; 3 Department of Radiotherapy, Benbadis University Hospital, Salah Boubnider University, Constantine, DZA; 4 Department of Surgery, Division of Radiation Oncology, Faculty of Medicine, University of British Columbia, Vancouver, CAN; 5 Department of Radiation Oncology, Ninevah University, Mosul, IRQ; 6 Department of Radiation Oncology, BC Cancer Centre for the North, Prince George, CAN

**Keywords:** academic radiation oncology, algeria, career development, general radiation oncology, global radiation oncology, medical practice, north africa, post-grad medical education, radiation oncology training, radiotherapy

## Abstract

Introduction

Radiation oncology is a dynamic and multifaceted specialty, essential in the multidisciplinary management of cancer. In Algeria, significant strides have been made in radiotherapy infrastructure and training since its independence in 1962. However, disparities in equipment distribution, limited access to continuing education, and workforce challenges persist. This study explores the training and career trajectories of Algerian radiation oncologists, highlighting the systemic and professional obstacles they face.

Methods

An anonymous national survey was conducted, targeting Algerian residents and specialists in radiation oncology. Of the 139 physicians invited, 35 responded (25% response rate).

Results

The results revealed that 34/35 (97% of respondents) believe current educational tools need to evolve to meet modern demands. Key concerns included insufficient clinical exposure during training, lack of access to mentorship, and geographic inequalities. A striking example is the public sector in Algiers (population >6 million) having the same number of linear accelerators (3) as the smaller city of Bechar (population ~350,000), highlighting a significant demographic imbalance, which leads to unequal access to treatment. Most participants identified radiobiology, radiology, radiotherapy physics, psychology, and side effect management as essential knowledge areas.

Conclusion

Algerian radiation oncologists advocate for updated curricula, formal mentorship, and international collaboration. To meet rising cancer demands, national strategies must prioritize standardized training, continuous education, and equitable resource allocation to ensure sustainable, high-quality care.

## Introduction

Radiation oncology is a unique specialty that involves multiple and complex activities, requiring diverse skills. It remains a rapidly evolving medical discipline driven by biological, clinical, and technological advancements [1]. Radiotherapy is recognized as one of the most essential approaches to cancer treatment, alongside surgery, chemotherapy, and, more recently, targeted therapy and immunotherapy. It is estimated that half of cancer patients will receive radiotherapy during their disease [2]. The radiation oncology team must support and guide cancer patients from diagnosis through treatment, provide long-term support for survivors, and offer end-of-life care for patients with limited life expectancy. Improvements in the safety of dose delivery to target volumes and the reduction of unnecessary but inevitable irradiation to organs at risk have increased the therapeutic ratio and the effectiveness of radiotherapy [3].

Radiation oncology training programs worldwide exhibit significant differences, primarily due to variations in the structure of their training [4]. The primary professional organization in each country (college or society) is responsible for designing the program and ensuring the transfer of skills between generations during radiation oncology training [4]. Current residency programs place a strong emphasis on theoretical and hands-on knowledge through training, radiotherapy conferences, and online webinars, though there is room to enhance standardized clinical exposure [5]. This is reflected in the fact that 97% of respondents expressed a need for updated educational tools (see Results). While initiatives like the Core Curriculum of the European Society for Radiotherapy and Oncology (ESTRO) provide valuable guidance for European training, not all regions, including Algeria, are yet integrated into such frameworks. This highlights an opportunity to further bridge gaps in mentorship and technological adoption to ensure equitable advancement in medical education. ESTRO plays a crucial role in maintaining and disseminating scientific advancements, as well as providing numerous educational activities. The "ESTRO Fellow" initiative represents the first attempt at harmonizing training standards across Europe [5].

Becoming a radiation oncologist in Algeria requires successfully passing the competitive entrance exam for specialized medical studies (residency), which is taken after obtaining a medical degree. The exam is organized by the Ministry of Higher Education and Scientific Research through a ministerial decree specifying the date, exam procedures, and the number of available positions (budgetary). The training lasts four years under the supervision of the department head and covers both theoretical and practical aspects, though much of the practical training is still based on 2D and 3D techniques, with limited exposure to modern advanced technologies.

Algeria's radiotherapy infrastructure has evolved significantly since independence, yet disparities persist due to centralized resource allocation and workforce shortages. With an aging population and rising cancer incidence, equitable access to radiotherapy remains a critical challenge. At independence in 1962, only three radiotherapy centers were operational in Algeria (North, Center-West, and East) for a population of 10 million, with two cobalt units, radium brachytherapy, and contact therapy. An ambitious program to establish cancer centers was launched by the state, considering the epidemiological context, cancer incidence, demand for radiotherapy care, and aging equipment. Radiotherapy was the first specialty taught in cancerology in 1985-1986 in Algiers and Constantine by Professors Ghouadni and Rahal (the first cohort in 1989 with three residents undergoing training abroad), which was before the medical oncology program that began in 1994. The early days involved simple machines, such as cobalt units, followed by first-generation linear accelerators that performed basic techniques and, eventually, the gradual acquisition of state-of-the-art accelerators offering innovative techniques nationwide.

Given the urgency and importance of continuing education for radiation oncologists, physicists, and radiotherapy technicians, the Association of Practitioners in Radiation Oncology (APOR) was established in 2012. The first APOR conference was held in Constantine from September 6 to 8, 2013, with the theme "Innovative Practices in Radiotherapy". To improve the quality of radiotherapy care for cancer patients, the National Cancer Plan 2015-2019 was developed, aiming to enhance the performance of radiotherapy services, reduce waiting times, improve the quality and safety of radiotherapy care, and strengthen training and education.

According to internal reports from the Algerian Ministry of Health used for strategic planning, beginning of the year 2025 [6], after years of installing high-quality equipment and transitioning from simple to innovative techniques, the number of linear accelerators has increased to 57 (12 in the private sector, 43 in the public sector, and two in the Ministry of Defense). Among the 40 accelerators in the public sector, nearly 50% are being renewed or are in the process of installation. Nationally, there are 22 radiotherapy centers in the public and military sectors, with 15 (68.18%) operational (three in the West, four in the East, one in the Center, four in the North, and three in the South) and seven centers set to open soon with 16 accelerators (West, Northwest, North, Center, and South). Additionally, there are six private centers, bringing the total to 70 accelerators by the end of 2025. Each radiotherapy department has one or two computed tomography (CT) scanners, with the possibility of virtual simulation in one center in Western Algeria. There are three gynecological brachytherapy units in public institutions (two in the Center and one in Western Algeria) and four private centers offering this service (one in the East, one in the West, and two in Central Algeria). Algeria is aware of the need to modernize its radiotherapy equipment and has implemented a second National Cancer Plan 2025-2030: strategic guidelines are being developed as part of ongoing efforts to combat cancer.

Low- and middle-income countries often have fragile health systems that are unable to respond to chronic health needs. Countries in crisis (e.g., Syria, Yemen, and rural areas of Africa) have radiotherapy equipment rates that are well below the World Health Organization (WHO) recommendations (e.g., fewer than one machine per 10 million people in some regions) [7]. Gulf countries (e.g., the United Arab Emirates) are investing in advanced equipment such as stereotactic radiotherapy (e.g., Gamma Knife®) and tomotherapy. Lebanon and Jordan have implemented pilot programs using mobile radiotherapy to reach rural populations and refugees [8].

According to data available on the website of the Directory of Radiotherapy Centers (DIRAC), which is part of the International Atomic Energy Agency (IAEA), 70% of countries without a single radiotherapy machine are in sub-Saharan Africa [9]. In this distribution of treatment machines, a noticeable disparity and inequality exist in the allocation of accelerators nationwide relative to the population and the number per million inhabitants in Algeria. This distribution highlights a significant geographic and demographic imbalance. For instance, the public sector in Algiers, the capital and a major referral hub with over six million inhabitants, operates only three linear accelerators. In contrast, Bechar, with a population of approximately 350,000, also operates three accelerators. This results in a dramatically lower number of machines per capita in the high-density capital region, leading to longer waiting times and potential inequity in access to care. Among these centers, only nine are training centers for radiation oncologists, while the others are led by young radiation oncologists who require guidance from senior colleagues. The radiation oncology community comprises 18 professors, many of whom head public departments, along with 54 senior assistants, 157 assistants, and 52 residents. Other physicians who completed their training have chosen to explore new horizons abroad after finishing their civil service, primarily citing better working conditions and attractive salaries. These risks lead to a shortage of radiation oncologists in some regions of Algeria. This number will need to increase due to the opening of new centers and the replacement of equipment in existing departments. The number of active senior professionals is 218 for 54 accelerators (70 by the end of 2025), resulting in a ratio of four physicians per machine at the time of writing. If new positions are not created, this ratio will drop to three, which remains reasonable with the implementation of optimal medical staff management (Appendix 1).

The radiotherapy sector in Algeria has significant development potential but faces several challenges, particularly in terms of human resources and infrastructure. However, its expansion faces major constraints such as regional disparities in access to equipped centers, delays in equipment renewal, overloading of professionals, brain drain of skilled specialists abroad, lack of an integrated network between the various centers, limited mentorship programs, financial constraints to international collaboration, and poor integration into clinical research.

Our study is the first to comprehensively analyze the training, career challenges, and systemic obstacles faced by radiation oncologists in Algeria, using a national survey. While prior research has explored similar themes in other regions (e.g., Italy, Japan, and Africa), our work uniquely highlights geographic disparities in resource allocation (e.g., Algiers vs. Bechar accelerator distribution); identifies Algeria-specific barriers, such as limited mentorship programs and financial constraints to international collaboration; and proposes localized solutions, including standardized curricula and diaspora engagement, tailored to Algeria's healthcare context. Additionally, we outline opportunities for improvement informed by practitioner experience, sustained political commitment, and enhanced collaboration among all sector stakeholders.

## Materials and methods

A questionnaire inspired by other experiences [4,10-13] was specifically designed to capture data on patient care, resident training, and the hopes and regrets of Algerian radiation oncologists, as well as their motivations and suggestions, to gain a general understanding of the challenges faced by our profession in Algeria. The survey instrument included questions on educational needs, working conditions, career regrets, and essential knowledge areas. A sample of key questions is presented below. We acknowledge the 25% response rate (35/139 participants). The participants are from various centers, regions, and career stages (residents, assistants, senior assistants, and professors), ensuring a balanced perspective. The survey is anonymous to reduce participation hesitancy. The excluded concerns incomplete surveys and non-physician staff. An invitation letter was sent on July 31, 2024, encouraging all physicians to participate in the questionnaire, and participants' consent was obtained before starting the survey via the survey's first page.

An anonymous online survey was conducted using the Google Forms platform (Google LLC, Mountain View, California, United States). The survey deadline was extended until October 23, 2024. The questionnaire consisted of a total of 15 objective questions (open-ended questions, ranking-based questions, and direct yes/no questions). Once the questionnaire was completed, responses were collected and analyzed anonymously. The results were analyzed statistically using descriptive methods based on the frequency of responses.

## Results

As shown in Table 1, 35 out of 139 physicians responded to the questionnaire (with a 25% response rate). Amidst the rapid advancements in medical technology, Algerian radiation oncology professionals voiced an almost unanimous call for change: 34 participants (97.1%) emphasized that modernizing educational tools was non-negotiable to keep pace with evolving practices. Nearly half (46.6%) of respondents noted that current systemic gaps could hinder training and clinical effectiveness, underscoring the importance of tailored solutions, as seen in Algeria's context. Residency positions frequently went unfilled (91.5%), trapped in a cycle of inadequate training facilities, poor awareness of the specialty's demands, and unsustainable work-life imbalances. To break this cycle, 88.6% championed a formal Maintenance of Certification (MOC) program, viewing continuous learning as vital for professional credibility and patient safety. Still, deeper gaps persisted. Four out of five respondents (80%) observed that future interns entered the field blind to the daily realities and lifestyle sacrifices of radiation oncology. While 91.4% lauded international forums like the American Society for Radiation Oncology (ASTRO) and ESTRO as indispensable for networking and innovation, and every single participant (100%) endorsed the value of ongoing education, 77.1% stressed a critical caveat: these resources must reach aspiring specialists before career commitments are made. When defining the role's core demands, technical mastery (94.1%) and medical knowledge (85.3%) dominated, though patient care skills (64.7%) remained a noted priority. Despite this collective drive for progress, a sobering undercurrent remained: over one-fifth (21.2%) of graduates rated their career regret at a palpable 7 out of 10, reflecting the personal toll of unmet challenges.

**Table 1:** Results of the 15 questions EU: European Union; MOC: Maintenance of Certification; ASTRO: American Society for Radiation Oncology; ESTRO: European Society for Radiotherapy and Oncology

Question	Response	Number (%)
(1) Given the technical advancements and rapid changes in patient care, to practice radiotherapy:		
A. Is it necessary to change educational tools?	Yes	34 (97.1)
	No	1 (2.9)
B. In 2024, will the challenges and evolving circumstances impact education and practice in our specialty?	Yes	30 (100)
	Yes with explanation	14 (46.6)
	No	0 (0)
(2) In the EU and across the Atlantic, residency positions remain vacant (less enthusiasm for the specialty). If this is the case in Algeria, list 1-3 main reasons.	Poor training conditions	32 (91.5)
	Unknown specialty	
	Work-life balance	
	Working conditions	
	Mandatory civil service	
	Lack of awareness of our specialty	
	Lack of support services	
(3) In the EU and across the Atlantic, the MOC program and certification boards emphasize continuous learning and professional development. What do you think about its application in Algeria for all categories?	Yes	31 (88.6)
	Yes with explanation	8 (22.9)
	No	4 (11.4)
(4) Do potential candidates for new residency positions have an understanding of the lifestyle and daily responsibilities? Orientation through documents or videos?	Yes	3 (8.6)
	No	28 (80)
	Other	4 (11.4)
(5) Do you think events like ASTRO, ESTRO, or, in their absence, local events are opportunities for networking, continuing education, and staying informed about the latest advances and innovations in radiation oncology?	Yes	31 (91.4)
	Yes with explanation	2 (5.7)
	Other	1 (2.9)
(6)A. Facing the challenges in radiation oncology in 2024, do you think resources and support systems are available to highlight career pathways?	Yes	32 (91.5)
	No	3 (8.5)
(6)B. Can continuing education and professional gatherings help radiation oncologists navigate their careers effectively?	Yes	35 (100)
	No	0 (0)
(7) If these resources are lacking, should their availability be required or hoped for before choosing this specialty?	Yes	27 (77.1)
	No	6 (17.1)
	Other	2 (5.7)
(8-9) The hopes and regrets of radiation oncologists can be diverse and deeply personal, often shaped by their experiences in training, practice, and interactions with patients.	The responses reflect concern for the physician's well-being, including their training, career, and professional status, as well as a desire to improve patient outcomes. The responses vary depending on the physician's position, priorities, and work-life balance. The discomfort, even significant concern, experienced during and after training is evident. This manifests when making therapeutic decisions due to insufficient clinical exposure, especially in managing complex situations.	29 (82.9)
(10) Do you agree with this conclusion?		
"The hopes and regrets of radiation oncologists reflect their commitment to patient care and the complexities of practicing in a demanding medical field. They strive for advancements in treatment and greater recognition of their role while grappling with the emotional and professional challenges inherent to oncology. Balancing these aspects remains a key element of their professional journey."	Yes	35 (100)
	No	0 (0)
(11) Hopes after obtaining the diploma:	The responses also reflect a certain anxiety about securing a place in the scientific community, hoping for opportunities to progress, conduct research, and publish.	
A. Career progression: advancement through academic positions and leadership roles or sub-specialization.		
B. Continuous learning, such as conferences and workshops.		
C. Building a patient-centered practice: prioritizing patients through communication, empathy, and education.		
D. Contributions to research: research and clinical trials.		
E. Networking and collaboration with established professionals, support, and knowledge sharing.		
(12) Regrets after obtaining the diploma		
A. Administrative burdens, paperwork, and detachment from clinical practice.	A	15 (42.9)
B. Financial pressures with low compensation.	B	31 (88.6)
C. Challenges related to work-life balance and professional and personal life.	C	19 (54.3)
D. Isolation in practice; working alone or in small groups.	D	17 (48.6)
E. Adaptation challenges; regrets about being unprepared for the transition.	E	9 (25.7)
The regrets here reflect the lack of preparation to transition effectively between training and taking on clinical and administrative responsibilities, all under financial pressure.		
(13) Do you agree with this conclusion?		
"The journey of training and obtaining the diploma for radiation oncologists is filled with hopes and regrets. Optimistic about making an impact on patients' lives, but the challenges produce feelings of regret. Through mentorship, support networks, effective training programs, and a focus on work-life balance, a more positive experience can be fostered for future radiation oncologists."	Yes	34 (97.1)
	No	1 (2.9)
(14) What knowledge is required to practice radiation oncology as a physician?	Medical knowledge	29 (85.3)
A. What medical knowledge is prerequisite or required?	Technical knowledge	32 (94.1)
B. What technical skills are prerequisites or required?	Patient care skills	22 (64.7)
C. What patient care skills are prerequisites or required?	Other knowledge	10 (29.4)
D. What other knowledge is prerequisite or required?	Evidence-based medicine	25 (71.5)
	Anatomy, radiology, physics, radiobiology, psychology	25 (71.5)
	Associations between radiotherapy and medical treatments	21 (60)
	Palliative care	20 (57.2)
(15) On a scale of 0-10, to what degree do you evaluate your regret after obtaining the diploma?	0	0 (0)
	1	6 (18.2)
	2	4 (12.1)
	3	3 (9.1)
	4	3 (9.1)
	5	7 (21.2)
	6	2 (6.1)
	7	4 (12.1)
	8	3 (9.1)
	9	1 (3)
	10	0 (0)

## Discussion

This study highlights persistent and interlinked challenges facing the radiation oncology profession in Algeria, encompassing training quality, working conditions, infrastructure, mentorship, and access to international scientific engagement. Nearly all respondents (34, 97%) underscored the urgent need for educational tools to keep pace with technological advancements, identifying radiobiology, imaging, psychology, and side effect management as essential competencies. Low remuneration reported by 31 participants (88.6%) and excessive administrative workload were also viewed as significant deterrents to professional satisfaction.

A particularly striking example of inequality is the geographic disparity in linear accelerator distribution. Algiers, with over six million inhabitants, operates only three linear accelerators, the same number as Bechar, which serves roughly 350,000 residents. This reflects broader inequities in healthcare access and reinforces the need for coordinated national planning in equipment allocation and maintenance.

Mentorship emerged as a key priority, with 31 participants (91.4%) calling for structured programs. While international examples, such as Italy's young Italian Association of Radiotherapy and Clinical Oncology (yAIRO) initiative, demonstrate the benefits of early-career engagement [3], Algeria faces both financial and linguistic barriers to implementing similar frameworks. The hybrid "Access to Care" (A2C) model, successfully deployed in Anglophone Africa, offers a scalable template for cost-effective training if adapted to Algeria's francophone context. Respondents also recognized the value of involvement in global societies such as ESTRO and ASTRO for access to guidelines, education, and annual congresses. However, prohibitive costs and limited institutional support significantly restrict participation.

Our findings parallel observations from surveys in Italy, Japan, and the United States, where insufficient curricular exposure to radiotherapy and inadequate integration of diagnostic imaging into training remain widespread issues. In Algeria, knowledge of diagnostic imaging is considered a prerequisite for practice, yet residency programs often lack structured onco-radiology curricula. Standardization, modeled on ESTRO's Core Curriculum, would address these deficiencies, align training with international benchmarks, and improve workforce mobility [12-15].

Lessons from the A2C program also point to the importance of fostering interdisciplinary collaboration, leadership capability, and shared decision-making in clinical practice. Algerian respondents frequently reported a lack of departmental leadership and resistance to teamwork, compounded by entrenched hierarchies that delay adoption of improvements. The absence of unified departmental strategies diverts attention from core priorities such as clinician education and patient-centered care [16,17].

Despite these barriers, participants expressed optimism that financial constraints could be mitigated through the strategic use of downtime, protocol development, and targeted training. They emphasized the need for training programs to integrate not only technical proficiency but also a broader understanding of oncology, patient care, and interdisciplinary collaboration. The majority favored increased clinical exposure, formalized mentorship, and consistent continuing education, echoing best practices from international models [18-22].

Mentorship, whether formal or informal, was recognized as a powerful driver of professional development, retention, and research productivity. In Algeria, it is currently practiced informally through supervisory relationships in clinical departments rather than structured programs. Evidence from initiatives such as the Radiation Oncology Academic Development and Mentorship Assessment Project (ROADMAP) program in the United States shows that formal mentor-mentee structures improve satisfaction, accelerate career advancement, and increase scholarly output. Establishing similar frameworks in Algeria could strengthen early-career retention and improve the specialty's profile nationally and internationally [12,23-26].

The findings also highlight Algeria's vulnerability to the growing cancer burden associated with an aging population. Meeting this challenge will require not only investment in new treatment centers but also ensuring that these facilities are staffed by professionals capable of safely commissioning and operating advanced technologies. Without parallel investment in comprehensive training and mentorship, the rapid expansion of infrastructure risks creating capacity without capability [27-30].

Addressing these priorities will not only enhance professional satisfaction and retention but also ensure equitable, high-quality radiotherapy access for all Algerian patients. The future of the specialty in Algeria will depend on the country's ability to couple infrastructure growth with human capital development, creating a balanced system capable of adapting to scientific advances while maintaining patient-centered care. Addressing the critical gaps in radiotherapy services demands a cohesive national strategy centered on sustainable development. The cornerstone of this effort is a comprehensive Curriculum Reform, introducing a standardized, competency-based national radiotherapy curriculum. This curriculum, modeled on ESTRO's respected Core Curriculum, will formally integrate essential onco-radiology modules to elevate foundational training. To bridge the transition from education to practice, structured mentorship programs need to be established nationally, pairing early-career professionals with experienced mentors, actively harnessing both local expertise and the valuable knowledge of the diaspora.

Ensuring skills remain current requires sustainable continuing education, funded through dedicated mechanisms to support lifelong learning via e-learning platforms, specialized masterclasses, and international exchanges. Concurrently, reliable service delivery hinges on proactive infrastructure and maintenance policies, mandating the integration of comprehensive equipment maintenance contracts into institutional budgets to prevent disruptive breakdowns. Finally, leveraging diaspora and international collaboration will be key; virtual platforms and visiting faculty programs will facilitate seamless expertise transfer and catalyze vital research partnerships, creating a resilient and interconnected radiotherapy ecosystem for the future.

The 25% response rate, while a common challenge in physician surveys, introduces a potential for selection bias, as respondents may be those more engaged or dissatisfied with the current system. This should be considered when generalizing the findings. Future studies could benefit from a mixed-methods approach, incorporating qualitative interviews to gain deeper insights into the challenges identified here.

## Conclusions

Algerian radiation oncologists operate within a complex environment of constrained resources, uneven access to care, and rapid technological change. While these challenges are significant, they are matched by a strong commitment among practitioners to improve patient outcomes, expand professional skills, and strengthen the specialty's future. By aligning training programs with international standards, formalizing mentorship, investing in continuing education, and strategically engaging with the Algerian diaspora, the country can develop a sustainable, skilled workforce capable of meeting the rising demand for cancer care.

## Appendices

Appendix 1

**Table 2:** Status of the existing radiotherapy services CHU: Centre Hospitalier Universitaire; CPMC: Centre Pierre Marie Curie; IMRT: intensity-modulated radiation therapy; HCA: Hôpital Central de l'Armée; VMAT: volumetric modulated arc therapy

Sector	Devices	Number	Functional	Observation
Public sector				
CHU Constantine	4 accelerators: 1 accelerator 18 MV, 2 accelerators 6 MV, 2 TrueBeam installed in 2024	1, 2, 1	3 accelerators	2 TrueBeam installed in 2024; upcoming renewal of the 3 accelerators
Sétif	3 accelerators	3	3 accelerators	Upcoming renewal of the 3 accelerators
Batna	3 accelerators	3	3 accelerators	Upcoming renewal of the 3 accelerators
Annaba	3 accelerators	3	3 accelerators	Upcoming renewal of the 3 accelerators
Ouargla	2 accelerators	2	2 accelerators	-
CPMC	3 accelerators	3	1 was renewed	Upcoming renewal of the 3 accelerators
Blida	3 accelerators: 1 accelerator 18 MV, 2 accelerators 6 MV	1, 2	Renewal of 1 Halcyon accelerator; the other 2 in progress	Upcoming renewal of 2 accelerators
El Oued	3 accelerators 18 MV	3	3 accelerators	-
Emir Abdelkader Oran	3 accelerators: 1 accelerator, 1 Halcyon, and 1 TrueBeam	1, 1, 1	3 accelerators	Renewal of 1 accelerator
Sidi Bel Abbès	3 accelerators 18 MV	3	Functional	3 accelerators with IMRT in operation
Tlemcen	3 accelerators 18 MV	3	Functional	3 accelerators with IMRT in operation
CHU Oran	2 accelerators	2	Commissioning	-
Adrar	3 accelerators 18 MV	3	Functional	2 accelerators are operational, but not the TrueBeam
Béchar	3 accelerators 18 MV	3	Functional	3 accelerators operational with IMRT
Draa Ben Khedda (Tizi Ouzou)	3 accelerators 18 MV	3	Functional	3 accelerators operational with IMRT
Djelfa	3 accelerators	3	Procedure in progress	-
Mostaganem	3 accelerators	3	Procedure in progress	-
Laghouat	3 accelerators	3	Procedure in progress	-
Chlef	3 accelerators	3	Procedure in progress	-
Algiers Beni Messous	2 accelerators	2	Procedure in progress	-
Algiers Rouiba	2 accelerators	2	Procedure in progress	-
HCA Algiers	2 accelerators: 1 accelerator 18 MV, 1 tomotherapy	1, 1	Functional	Functional with IMRT
Private sector				
Clinique Athena	2 accelerators 18 MV	2	Functional	Functional with VMAT
Clinique Sidi Abdellah	2 tomotherapy units	2	Functional	Functional
Clinique Khodja Bach	2 accelerators 18 MV	2	Functional	Functional with IMRT
Tizi Ouzou Mahmoudi	2 accelerators 18 MV	2	Functional	Functional with IMRT and stereotactic radiotherapy
Clinique Blida	2 accelerators	2	Functional	Functional
Private Clinic Oncopole Oran	2 accelerators 18 MV	2	Functional	Functional

## References

[1] Pötter R, Eriksen JG, Beavis AW, Coffey M, Verfaillie C, Leer JW, Valentini V (2012). Competencies in radiation oncology: a new approach for education and training of professionals for radiotherapy and oncology in Europe. Radiother Oncol.

[2] Baskar R, Lee KA, Yeo R, Yeoh KW (2012). Cancer and radiation therapy: current advances and future directions. Int J Med Sci.

[3] Nardone V, Boldrini L, Salvestrini V, Greco C, Petrianni GM, Desideri I, De Felice F (2023). Are you planning to be a radiation oncologist? A survey by the young group of the Italian Association of Radiotherapy and Clinical Oncology (yAIRO). Radiol Med.

[4] Mula-Hussain L, Wadi-Ramahi S, Li B, Ahmed S, De Moraes FY (2021). Specialty Portfolio in Radiation Oncology: A Global Certification Roadmap for Trainers and Trainees (Handbook - Logbook). https://qspace.qu.edu.qa/xmlui/handle/10576/17692.

[5] Turner S, Eriksen JG, Trotter T (2015). Establishing a global radiation oncology collaboration in education (GRaCE): objectives and priorities. Radiother Oncol.

[6] (2025). Algeria intensifies its fight against cancer through a coordinated strategy. https://www.horizons.dz/?p=271500.

[7] Alawa J, Coutts A, Khoshnood K (2021). Cancer care in low- and middle-income countries affected by humanitarian crises. Handbook of Healthcare in the Arab World.

[8] (2022). Cancer in the Arab world.

[9] (2025). Expanding global access to radiotherapy - pragmatic steps for consideration in providing cancer care in sub-Saharan Africa. https://www.estro.org/ESTRO/media/ESTRO/About/Newsletters/Make%20it%20happen/Expanding-Global-Access-to-Radiotherapy-%E2%80%93-pragmatic-steps-for-consideration-in-providing-cancer-care-in-sub-Saharan-Africa_Make-It-Happen-Corner.pdf.

[10] Rodríguez A, Arenas M, Lara PC (2019). Are there enough radiation oncologists to lead the new Spanish radiotherapy?. Clin Transl Oncol.

[11] Napieralska A, Tomasik B, Spałek M, Chyrek A, Fijuth J (2021). Radiation oncology training in Poland: multi-institutional survey. J Cancer Educ.

[12] Wang MH, Loewen SK, Giuliani M, Fairchild A, Yee D, Debenham BJ (2022). Clinical learning, didactic education, and research experiences of radiation oncology resident physicians in Canada. J Cancer Educ.

[13] Murakami Y, Noda SE, Hatayama Y, Maebayashi T, Jingu K, Nagata Y, Mizowaki T (2020). What motivated medical students and residents to become radiation oncologists in Japan?-Questionnaire report by the radiotherapy promotion committee of JASTRO. J Radiat Res.

[14] Mukherjee A, Manir KS, Basu P, Mallik S, Goswami J (2021). Choosing a career in radiation oncology in India: a survey among young radiation oncologists. J Cancer Res Ther.

[15] Goodman CR, Sim AJ, Jeans EB (2021). No longer a match: trends in Radiation Oncology National Resident Matching Program (NRMP) data from 2010-2020 and comparison across specialties. Int J Radiat Oncol Biol Phys.

[16] Matalon SA, Howard SA, Abrams MJ (2019). Assessment of radiology training during radiation oncology residency. J Cancer Educ.

[17] Burger H, Wyrley-Birch B, Joubert N (2022). Bridging the radiotherapy education gap in Africa: lessons learnt from the Cape Town Access to Care Training Programme over the past 5 years (2015-2019). J Cancer Educ.

[18] Leer JW (2011). The future development of radiation oncology in Europe: a personal view. Radiother Oncol.

[19] Overgaard J (2011). Advancing radiation oncology through scientific publication - 100 volumes of radiotherapy and oncology. Radiother Oncol.

[20] Rodemann HP, Wouters BG (2011). Frontiers in molecular radiation biology/oncology. Radiother Oncol.

[21] Thwaites DI, Malicki J (2011). Physics and technology in ESTRO and in radiotherapy and oncology: past, present and into the 4th dimension. Radiother Oncol.

[22] Rosenblatt E, Zubizarreta E, Wondergem J, Fidarova E, Izewska J (2012). The International Atomic Energy Agency (IAEA): an active role in the global fight against cancer. Radiother Oncol.

[23] Benstead K, Lara PC, Andreopoulos D (2019). Recommended ESTRO Core Curriculum for Radiation Oncology/Radiotherapy 4th edition. Radiother Oncol.

[24] Baumann M, Leer JW, Dahl O, De Neve W, Hunter R, Rampling R, Verfaillie C (2004). Updated European core curriculum for radiotherapists (radiation oncologists). Recommended curriculum for the specialist training of medical practitioners in radiotherapy (radiation oncology) within Europe. Radiother Oncol.

[25] Eriksen JG, Beavis AW, Coffey MA (2012). The updated ESTRO core curricula 2011 for clinicians, medical physicists and RTTs in radiotherapy/radiation oncology. Radiother Oncol.

[26] Sambunjak D, Straus SE, Marusić A (2006). Mentoring in academic medicine: a systematic review. JAMA.

[27] Holliday EB, Jagsi R, Thomas CR Jr, Wilson LD, Fuller CD (2014). Standing on the shoulders of giants: results from the Radiation Oncology Academic Development and Mentorship Assessment Project (ROADMAP). Int J Radiat Oncol Biol Phys.

[28] Efstathiou JA, Drumm MR, Paly JP (2018). Long-term impact of a faculty mentoring program in academic medicine. PLoS One.

[29] Croke J, Tosoni S, Ringash J (2021). "It's good for the soul:" perceptions of a formal junior faculty mentorship program at a large academic cancer centre. Radiother Oncol.

[30] Lin D, Gomez DR, Zhang YH (2022). Radiation Oncology AcaDemic Mentorship Program (ROADMAP) for junior faculty: one-year results of a prospective single institution initiative. Int J Radiat Oncol Biol Phys.

